# Predicting susceptibility to inflammaging using a genetic inflammatory index through Prakriti-based phenotypic stratification

**DOI:** 10.3389/fimmu.2026.1854940

**Published:** 2026-07-29

**Authors:** Neha Bera, Agaje Gowri S, Nousheen Syed, Varadhi Govinda, A. Karteek Rao, Jahnavi Jeeru, Krishna Kurthkoti, Kamatham Narayanaswamy, Surendar R. Jakka, Akhouri Kishore Raghawan, Pratibha Nair, Baswanth Oruganti

**Affiliations:** 1 School of Biosciences, Chanakya University, Bengaluru, Karnataka, India; 2 Department of Chemistry, SRM University-AP, Amaravati, Andhra Pradesh, India; 3 Department of Organic Chemistry, Gayatri Vidya Parishad College for Degree and PG Courses (A), Visakhapatnam, Andhra Pradesh, India; 4 Department of Chemistry, GITAM Institute of Science, GITAM (deemed to be University), Visakhapatnam, Andhra Pradesh, India; 5 Department of Inorganic and Physical Chemistry, Indian Institute of Science, Bengaluru, Karnataka, India; 6 Dana-Farber Cancer Institute, Boston, MA, United States; 7 Department of Kayachikitsa, VPSV Ayurveda College, Kottakkal, Kerala, India; 8 Department of Mathematics and Mathematical Statistics, Umea˚ University, Umea, Sweden

**Keywords:** ayurgenomics, ayurveda, epigenetic age acceleration, immunometabolic reprogramming, trained immunity

## Abstract

Inflammaging is chronic low-grade inflammation arising from a progressive imbalance between pro-inflammatory and anti-inflammatory immune networks. The rate of inflammaging exhibits marked interindividual variation owing to differences in the pace of biological aging. Within the two-hit theory of inflammaging, such variability involves both genetic and epigenetic components. Stratification models derived from quantification of the genetic component allow classification of individuals according to their baseline genetic inflammatory tone. Such phenotypic stratification could facilitate informed decision-making in prioritizing medical care and vaccination strategies during public healthcare emergencies, including epidemics and pandemics. To this end, this paper develops a novel conceptual framework that predicts susceptibility to inflammaging based on an individual’s unique constitutional phenotype, termed Prakriti. Based on the Prakriti-based phenotypic stratification rooted in Ayurveda (Indian traditional medicine) and emerging ayurgenomics evidence suggesting genotype-phenotype correlations, we hypothesize that Prakriti constitutes a phenotypic biomarker of genetic inflammatory tone. Specifically, we introduce a mathematical model that quantifies the baseline inflammatory tone as the Genetic Inflammatory Index (GII), a measure of constitutional inflammatory architecture derived from cytokine single nucleotide polymorphism (SNP) scores. Based on the sign of GII, we hypothesize that Vata and Pitta dominant constitutions carry a pro-inflammatory (GII *>* 0) genetic predisposition, while Kapha dominant constitutions are hypothesized to possess an anti-inflammatory (GII *<* 0) genetic resilience. Accordingly, within age-matched cohorts exposed to broadly comparable environmental conditions, Vata and Pitta Prakriti individuals are predicted to exhibit greater epigenetic age acceleration than Kapha Prakriti individuals. Finally, we outline experimental strategies to test these hypotheses using epigenetic clocks in Prakriti-stratified cohort studies.

## 1 Introduction

During the COVID-19 pandemic, one issue that garnered substantial interest was identifying risk factors for progression of the COVID-19 disease to critical stages that involves hyperinflammatory immune response against the infection, termed as cytokine storm. While aging and age-related chronic diseases such as type 2 diabetes (T2D), obesity, hypertension, atherosclerosis, cancer, cardiovascular and autoimmune diseases were identified as some key risk factors (1, 2), it has become clear that the underlying mechanism that interconnects such chronic diseases with COVID-19 pathogenesis is a process known as inflammaging (3–7). Inflammaging is an increased chronic low-grade inflammatory state of the innate immune system that occurs with age, primarily characterized by an imbalance between production of pro-inflammatory and anti-inflammatory cytokines by the innate immune system. Counteracting such imbalance facilitates longevity in centenarians, which has been associated not with the absence of pro-inflammatory cytokines but with a dynamic balance between elevated IL-6, TNF-*α*, and IFN-*α* and compensatory increases in anti-inflammatory mediators such as IL-19 (8).

**Figure 1:**
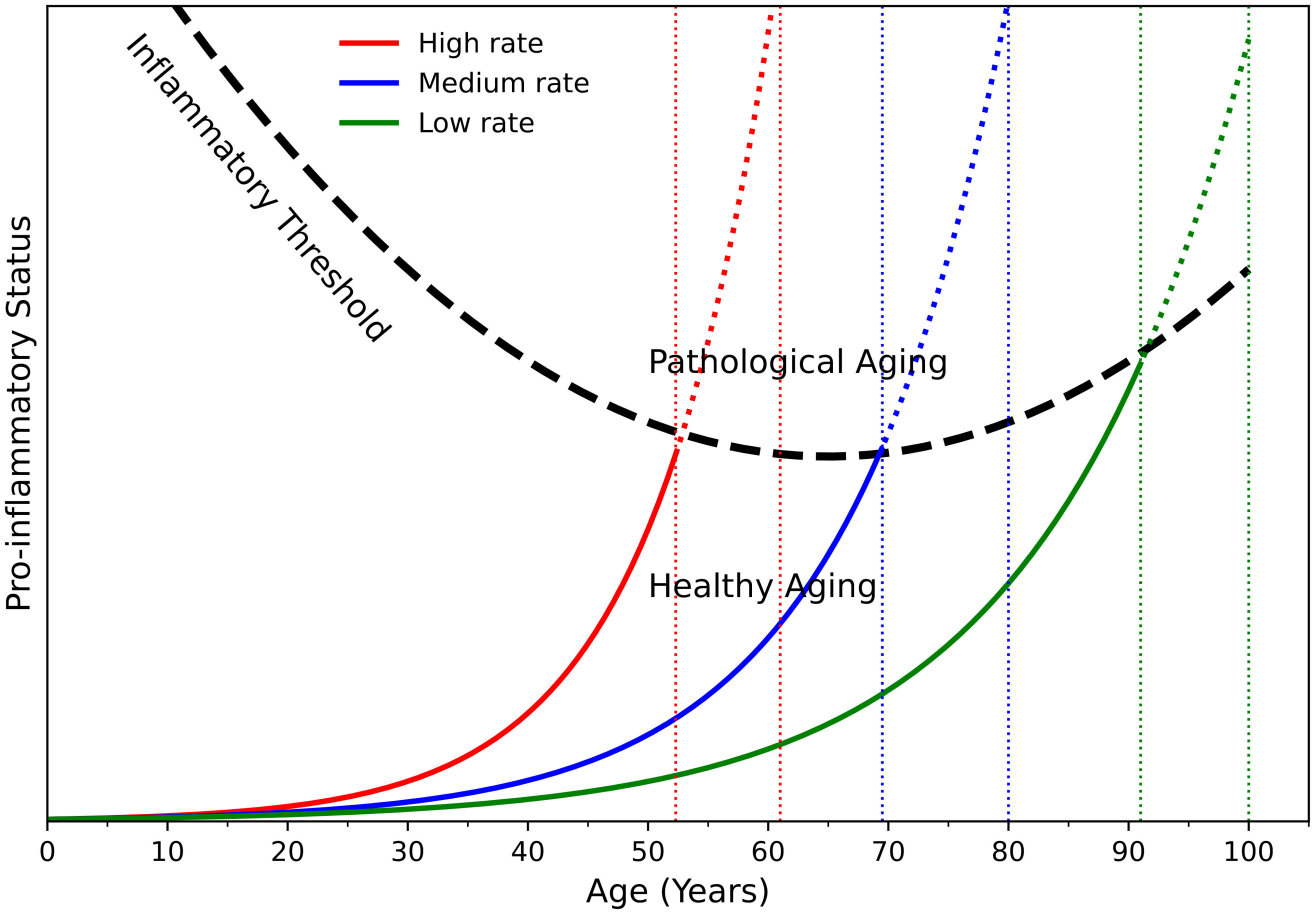
The inflammaging threshold model proposed by Franceschi et al. (4). The rate at which an individual reaches the pro-inflammatory threshold — determined by genetic makeup and cumulative exposure to inflammatory stimuli — predicts susceptibility to chronic age-related diseases and dictates lifespan. Individuals with frail gene alleles and high inflammatory exposure reach the threshold earlier (accelerated aging), while those with robust alleles and low exposure age more slowly. The plots were generated using hypothetical data following the inflammaging threshold model by Franceshi et al. (4).

The term inflammaging was first coined by Claudio Franceschi et al. (4). Inflammaging constitutes a threshold in the pro-inflammatory state associated with pathological aging, the rate of reaching which is governed by two factors: the absence of robust gene alleles or presence of frail gene alleles, and persistent cumulative exposure to inflammatory stimuli over time (3, 4). Consequently, individuals possessing frail gene alleles or those exposed to a high inflammatory burden are expected to exhibit increased susceptibility to pathological aging and age-related chronic diseases, as illustrated in **Figure 1**. Inflammaging can therefore be regarded as a manifestation of accelerated aging that reduces lifespan. The emerging field of geroscience accordingly aims to counteract age-related diseases collectively, by identifying and targeting fundamental mechanisms of inflammaging rather than addressing individual diseases in isolation.

Understanding and predicting interindividual variation in susceptibility to chronic age-related diseases is a central theme of P4 medicine — predictive, preventative, personalized, and participatory medicine (9, 10). P4 medicine rests on the premise that each person’s health is unique and that uniform approaches to prevention and treatment are inherently inadequate. Two prominent frameworks within P4 medicine are pharmacogenomics (11, 12) and ayurgenomics (13–15). Pharmacogenomics quantifies interindividual variation in drug responses as a function of genetic polymorphism in drug-metabolizing and transporting enzymes. Ayurgenomics, in contrast, is primarily concerned with predicting susceptibility to chronic age-related diseases as a function of phenotypic variation — termed Prakriti in Ayurveda — by correlating it with genotypic variation (13–15). Ayurveda is a system of personalized traditional medicine practiced in India for over 3,500 years that classifies individuals into primarily three Prakriti types — Vata, Pitta, and Kapha — characterized by specific phenotypic attributes known as Gurvadi Gunas. These attributes act as foundational parameters that manifest as observable variations in metabolism (Agni) and immune resilience (Ojas).

The present hypothesis paper integrates insights from inflammaging, ayurgenomics, and Ayurveda to develop a conceptual framework for predicting susceptibility to inflammaging through a genotype-Prakriti correlation in inflammation. By proposing a mathematical model quantifying cumulative single nucleotide polymorphisms (SNPs) in genes associated with pro-inflammatory and anti-inflammatory cytokines, we derive a Genetic Inflammatory Index (GII) that correlates interindividual variations in baseline genetic inflammatory tone with differences in Prakriti in Ayurveda. Specifically, we hypothesize that distinct Prakriti types exhibit systematic differences in baseline inflammatory tone, with Vata and Pitta individuals being more susceptible to inflammaging than Kapha, which can be validated in terms of differences in epigenetic age acceleration.

The paper is organized as follows. Section 2 discusses the causes and stimuli that drive inflammaging, the two key mechanisms of inflammaging: epigenetic and immunometabolic reprogramming, and epigenetic clocks that quantify these changes. Section 3 presents the concept of Prakriti in Ayurveda, and discusses the Ayurvedic perspective on aging. Section 4 integrates these insights into a set of novel hypotheses connecting Prakriti and inflammaging, and Section 5 outlines experimental strategies to test the hypotheses, and the final section presents an integrated discussion, and identifies limitations of the proposed model.

## 2 Causes, mechanisms and biomarkers of inflammaging

### 2.1 Inflammatory stimuli and processes

**Figure 2:**
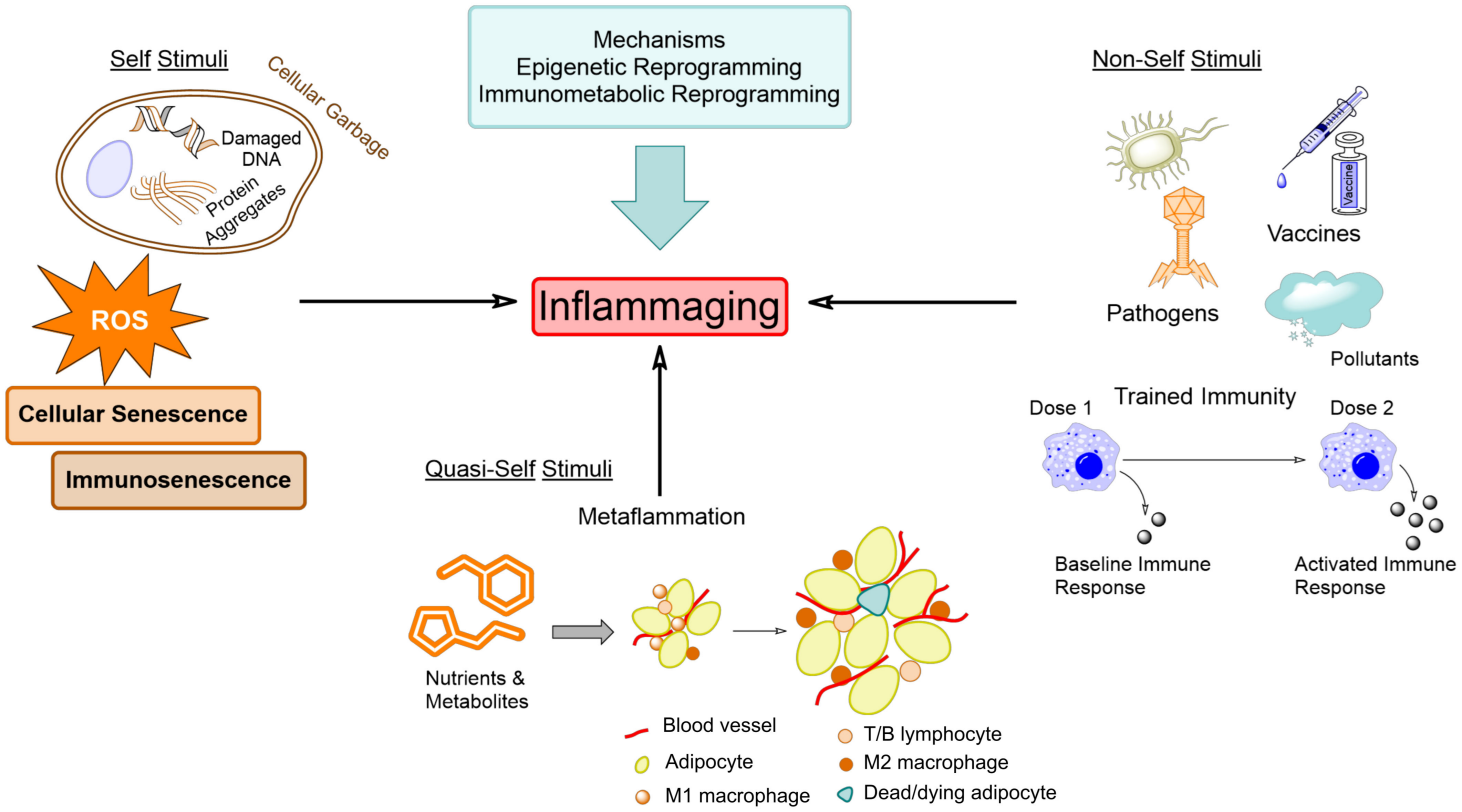
Stimuli, processes, and mechanisms of inflammaging. Self, non-self, and quasi-self stimuli contribute to inflammaging through induction of cellular senescence, immunosenescence, trained immunity, and metaflammation. Self-stimuli include cellular garbage such as damaged DNA, protein aggregates, and reactive oxygen species (ROS); non-self stimuli include pathogens, vaccines, and environmental pollutants; whereas quasi-self stimuli arise from metabolic perturbations associated with nutrient excess causing adipocyte hypertrophy and inflamed adipose tissue. These processes are regulated by two central mechanisms, namely epigenetic and immunometabolic reprogramming.

Stimuli that drive inflammaging can be broadly classified into three categories: self (endogenous), quasi-self, and non-self (exogenous), all of which are interconnected in a complex interplay between two mechanisms: epigenetic and immunometabolic reprogramming, as illustrated in **Figure 2**. Some key processes that contribute to or regulate inflammaging include oxidative stress (16–18), cellular senescence (19, 20), immunosenescence (21, 22), metaflammation (23–26), and trained immunity (27–30), many of which span more than one category. However, in the following discussion, for the sake of brevity of presentation, we categorize each process under only one of the classes and limit our discussion to those processes that have been extensively studied in the context of inflammaging.

#### 2.1.1 Self stimuli and related processes

##### 2.1.1.1 Cellular garbage, oxidative stress, and cellular senescence

Cellular garbage refers to a wide range of cellular components including damaged DNA, misfolded proteins, dysfunctional mitochondria or organelles, and other cellular components that are no longer functioning properly. These stressors constitute damage-associated molecular patterns (DAMPs) that are recognized by pattern recognition receptors (PRRs), such as Toll-like receptor (TLRs) and NOD-like receptors (NLRs), of the innate immune cells. The accumulation of cellular garbage stimulates the production of reactive oxygen species (ROS) (19, 20), including peroxides and free radicals, which contribute to cellular damage and may eventually cause cell death. Oxidative stress occurs when there is an imbalance between the production of ROS and the body’s antioxidant defense mechanisms in neutralizing them to prevent cellular damage. Autophagy and mitophagy are essential self-repair mechanisms that clear cellular garbage and maintain cellular homeostasis. With aging, the production of cellular garbage increases, while the efficiency of these mechanisms decreases. Furthermore, the expression of antioxidant enzymes, such as glutathione peroxidase, superoxide dismutase and catalase, diminishes with age (17). This cumulative dysfunction leads to impaired clearance of cellular garbage and sustains oxidative stress, which triggers the activation of the NLR family pyrin containing domain 3 (NLRP3) inflammasome (17, 31). Upon activation, the NLRP3 inflammasome promotes the activation of caspase-1, resulting in the maturation and release of pro-inflammatory cytokines, including IL-1*β* and IL-18 (31), which exacerbate tissue damage. Oxidative stress also triggers cellular senescence, a state in which cells are no longer able to divide and function normally. Senescent cells exhibit a unique secretory phenotype (SASP) (32, 33), which promotes inflammation through the production of pro-inflammatory cytokines such as IL-6, IL-1*β*, and TNF-*α*, chemokines, growth factors, and proteases (32, 33).

##### 2.1.1.2 Immunosenescence

Immunosenescence is a remodeling of the functions of the immune system with age, leading to ineffective or delayed responses against pathogens, and reduced response to vaccination in older individuals (21, 22, 34). While inflammaging involves increased pro-inflammatory activity of the innate immune cells, such as monocytes and macrophages, immunosenescence is characterized by a decrease in the effector functions of these cells, such as phagocytosis, free-radical production, chemotaxis, and antigen-presenting capacity (21, 22, 34, 35). Although an initial anti-inflammatory burst is triggered in response to the pro-inflammatory activity, it is often not sustained or is inefficient in older individuals to completely resolve the inflammation. Overall, inflammaging and immunosenescence collectively sustain an increased baseline threshold level of inflammation in the elderly to ensure that innate immune system remains alert against any serious pathogen challenge. It was suggested that immunosenescence might be a consequence of the energetic costs associated with maintenance of high intensities in both pro-inflammatory activity and in effector functions in older individuals (22). As a result, effector functions are compromised at the cost of maintaining high innate immune cell alertness. Such a remodeling or adaptation in innate immune functions might be essential to cope with other age-associated changes in the body to ensure long-term survival (22, 34).

#### 2.1.2 Quasi-self stimuli and related processes

##### 2.1.2.1 Nutrients and metaflammation

Metaflammation is a state of chronic low-grade inflammation driven by long-term nutrient excess or over-nutrition, observed commonly in endocrine-metabolic disorders such as obesity and T2D (24, 25, 36, 37). Similar to senescent cells, nutrients present in nutrient-dense or high-fat diets act as DAMPs, and are recognized by TLRs of adipocytes, such as TLR4, leading to the production of pro-inflammatory cytokines such as IL-6 and TNF-*α* (23, 24). Such increase in inflammatory responses upon ingestion of food is termed as post-prandial inflammation. Repeated cycles of post-prandial inflammation might cause adipocyte hypertrophy, which causes low responsiveness to insulin and increase in the release of free fatty acids into the blood. Moreover, hypertrophic adipocytes undergo apoptosis, which leads to the recruitment of immune cells, such as macrophages, to the adipose tissue (AT), which further exacerbates the pro-inflammatory environment of the AT. Thus, metaflammation can be considered as nutrient-excess driven accelerated aging, and metabolic disorders can be thought of as manifestations of metaflammation.

#### 2.1.3 Non-self stimuli and related processes

Non-self stimuli include pathogens, pollutants, allergens, and toxins, which possess specific epitopes or patterns, known as pathogen associated molecular patterns (PAMPs), that are recognized by PRRs, such as TLRs or NLRs, of innate immune cells. This recognition triggers acute inflammatory response, leading to production of a variety of cytokines and chemokines by innate immune cells. However, interindividual variability in acute inflammatory response can occur due to factors such as pre-existing chronic inflammatory conditions or involvement of trained immunity (38, 39).

##### 2.1.3.1 Interplay between acute and chronic inflammation

When an initial acute inflammatory response is sufficient to eliminate a stressor, inflammation subsides due to the release of anti-inflammatory mediators without any involvement of the adaptive immune system. But more commonly, acute inflammation is terminated only after the activation of the adaptive immune system via antigen presentation by innate immune cells, such as macrophages and neutrophils, to naive T-cells. However, as discussed above, immunosenescence diminishes antigen presentation capacity and associated differentiation of naive Tcells into effector T-cells (21, 22), thereby reducing the effectiveness of adaptive immune system in resolving inflammation. This eventually results in chronic inflammation contributing to the development of inflammaging and chronic age-related diseases (21, 22). On the other hand, existing chronic inflammatory conditions can accentuate the acute inflammatory response against an invading pathogen, and transform it into a hyperinflammatory response leading to cytokine storm, as observed in severe COVID-19 disease (40). Thus, interplay between acute and chronic inflammation, and inefficiency of counteracting anti-inflammatory networks are key factors in predicting an individual’s susceptibility to age-related chronic diseases.

##### 2.1.3.2 Trained immunity and immunobiography

Trained immunity is a memory-like feature displayed by the innate immune system, resulting in a rapid and enhanced immune response upon a secondary infection (28–30). Innate immune memory can also manifest as a diminished immune response upon a secondary infection, referred to as innate immune tolerance (41). A unique aspect of these two processes is their heterologous nature, wherein enhanced or diminished responses are observed also against pathogens or stimuli that are unrelated to any previous insults. This cross protection is found to be independent of T- and B-lymphocytes of the adaptive arm. Rather, the protective effects are due to enhanced expression of PRRs of innate immune cells and associated increased production of pro-inflammatory cytokines due to epigenetic changes (41).

The concept of trained immunity emerged from studies on non-specific protective effects offered by Bacille Calmette-Guerin (BCG) vaccine against unrelated pathogens (42–44). Specifically, BCG vaccination in healthy individuals was found to modulate the phenotype of circulating monocytes, resulting in enhanced pro-inflammatory cytokine production. Notably, a 4–7 fold increase in the production of IFN-*γ*, and a two-fold increase in TNF-*α* and IL-1*β* levels were observed. Additionally, it was found that BCG vaccine amplifies pro-inflammatory activity of natural killer (NK) cells against *Candida Albicans* (42), through increased cytokine production and cytotoxic activity, even three months after vaccination, and has also been shown to be effective in preventing infection by an attenuated yellow virus vaccine strain (44). The protective effects associated with trained immunity are mediated by phenotypic changes in innate immune cells, such as monocytes, macrophages and NK cells (44, 45).

Epigenetic changes induced by trained immunity can be sustained over an extended period due to persistent exposure to similar types of pathogens, pollutants, toxins, nutrients, cultural habits, and so on, all of which constitute the immunobiography of an individual (39, 46). Understanding an individual’s immunobiography is important in predicting susceptibility to pathogenic infections as well as to age-related chronic diseases. For example, an individual who has been exposed to a variety of pathogens or stimuli over lifetime might have a more diverse and robust immune system that is better equipped to fight off future infections. In contrast, it might lead to a gradual increase of inflammatory tone, resulting in an increased baseline inflammation levels over time, contributing to inflammaging and its deleterious effects (29, 38, 47).

### 2.2 Mechanisms and biomarkers

Diverse processes that contribute to inflammaging described above converge on two deeply interconnected molecular mechanisms: epigenetic reprogramming and immunometabolic reprogramming. These mechanisms operate across multiple biological contexts—including cellular senescence, metaflammation, and trained immunity —and together establish a self-reinforcing pro-inflammatory state that intensifies with age.

### 2.3 Epigenetic reprogramming

Epigenetic reprogramming refers to changes induced in gene expression caused by covalent modifications in DNA or post-translational modifications in histones, without any alterations in the underlying DNA sequence. Examples of epigenetic modifications include DNA methylation, histone acetylation, and noncoding RNA-mediated gene regulation. These processes regulate chromatin accessibility and thereby control transcriptional activity of inflammatory genes in response to environmental and cellular signals.

#### 2.3.1 Epigenetic reprogramming in cellular senescence

Epigenetic mechanisms are central in cellular senescence as senescent cells undergo large-scale chromatin reorganization characterized by the formation of senescence-associated heterochromatin foci (SAHF), which repress proliferation-associated genes through repressive marks such as H3K9me3 and H3K27me3 (48, 49). A key feature of this reorganization is the functional separation between chromatin domains governing growth arrest and those driving the SASP (32, 33). High-mobility group (HMG) proteins orchestrate this partitioning: HMGA1 and HMGA2 support SAHF formation, while HMGB2 maintains SASP gene loci in an open, transcriptionally active state (48, 50). This selective chromatin accessibility enables sustained expression of pro-inflammatory mediators such as IL-6, IL-8, and TNF-*α* despite global heterochromatinization (48). Interestingly, it was observed that sirtuin1 (SIRT1), an NAD+-dependent deacetylase, negatively regulates the expression of SASP factors by binding to promoter regions of IL-6 and IL-8 through deacetylation of H3K9 and H3K16 (51).

#### 2.3.2 Epigenetic reprogramming in metaflammation

Epigenetic reprogramming plays a central role in metaflammation, particularly in T2D and AT inflammation in obesity. TLRs and TNF-*α* receptor (TNFR) recognize nutrients such as saturated free fatty acids as DAMPs and induce the production of pro-inflammatory markers including cytokines and transcription factors such as nuclear factor *κ*B (NF-*κ*B), which in turn drive transcription of pro-inflammatory genes such as TNF-*α* and IL-6 to propagate the inflammatory profile (52). Importantly, this inflammatory activation is not merely transcriptional — it is encoded epigenetically through differential DNA methylation of metabolic and cytokine gene promoter regions. Increased DNA methyltransferase 1 (DNMT1) expression at the adiponectin gene promoter region is observed in adipocytes of obese subjects, correlating negatively with adiponectin expression — indicating that DNMT1-mediated hypermethylation actively suppresses this key insulin-sensitising and anti-inflammatory adipokine in the obese state (53). Beyond the suppression of anti-inflammatory mediators, increased gene expression of pro-inflammatory cytokines TNF-*α* and MCP-1 was observed in transgenic mice overexpressing DNMT3a (54). Evidence from individual cytokine gene loci further supports the role of pro-inflammatory epigenetic reprogramming. Hypomethylation was observed in the TNF-*α* gene promoter region in peripheral white blood cells in women with higher truncal fat, correlating with higher TNF-*α* levels in plasma (55). Similarly, hypomethylation of the IL-1*β* promoter gene, and concomitantly increased IL-1*β* plasma levels, was found in peripheral blood mononuclear cells of T2D patients (56). A systematic study of 94 inflammatory genes in visceral AT found that 28 were differentially methylated in obesity, with 10 showing hypomethylation and increased expression of pro-inflammatory cytokines including IL-1*β*, IL-6, IL-8, IL-17, TNF-*α*, and IFN-*γ* (57).

#### 2.3.3 Epigenetic reprogramming in trained immunity

Epigenetic reprogramming in trained immunity has been extensively studied in the context of BCG vaccination. The enhanced production of pro-inflammatory cytokines such as TNF-*α* and IL-1*β* in BCG vaccinated individuals results from increased transcription driven by phenotypic changes in circulating monocytes, reflected in elevated mRNA expression for these cytokines (42). Mechanistically, these changes are mediated by increased H3K4 trimethylation at promoter regions of associated pro-inflammatory genes, providing a direct epigenetic basis for the enhanced inflammatory tone (42). Beyond the short-term phenotypic changes of circulating monocytes, the durability of BCG-induced trained immunity extends to hematopoietic stem cells (HSCs). BCG reprograms the transcriptional landscape of HSCs to generate epigenetically modified macrophages, resulting in a relatively long-lived pro-inflammatory tone (43), which adds to the cumulative inflammatory load and may contribute to deleterious effects of trained immunity in the long-term (29). The constitutional determinants of trained immunity amplitude were systematically investigated by Moorlag et al. in a comprehensive study of interindividual variation in innate immune responses at baseline and upon *in vivo* stimulation with four microbial stimuli: *Candida albicans*, *Escherichia coli lipopolysaccharide*, *Staphylococcus aureus*, and *Mycobacterium tuberculosis* (58). They identified a baseline epigenetic signature that determines the amplitude of trained immunity.

Beyond the epigenetic landscape, genome wide analysis studies identified strong associations of IL-1*β* and TNF-*α* production after *Candida Albicans* stimulation with a genetic variant (rs7136826) in a locus affecting the expression of PRRs involved in antifungal immunity (58). Moreover, interindividual variation in BCG-induced cytokine production capacity showed stronger correlation with differences in genotype and chromatin accessibility profiles at baseline than with any other measured variable — suggesting that constitutional genetic architecture is an important factor in trained immunity amplitude (58). These observations pose an interesting question: can baseline genetic differences in trained immunity amplitude be captured by differences in constitutional phenotype (Prakriti) in Ayurveda, and do they translate to variation in inflammatory load and differential susceptibility to inflammaging?

### 2.4 Immunometabolic reprogramming

**Figure 3:**
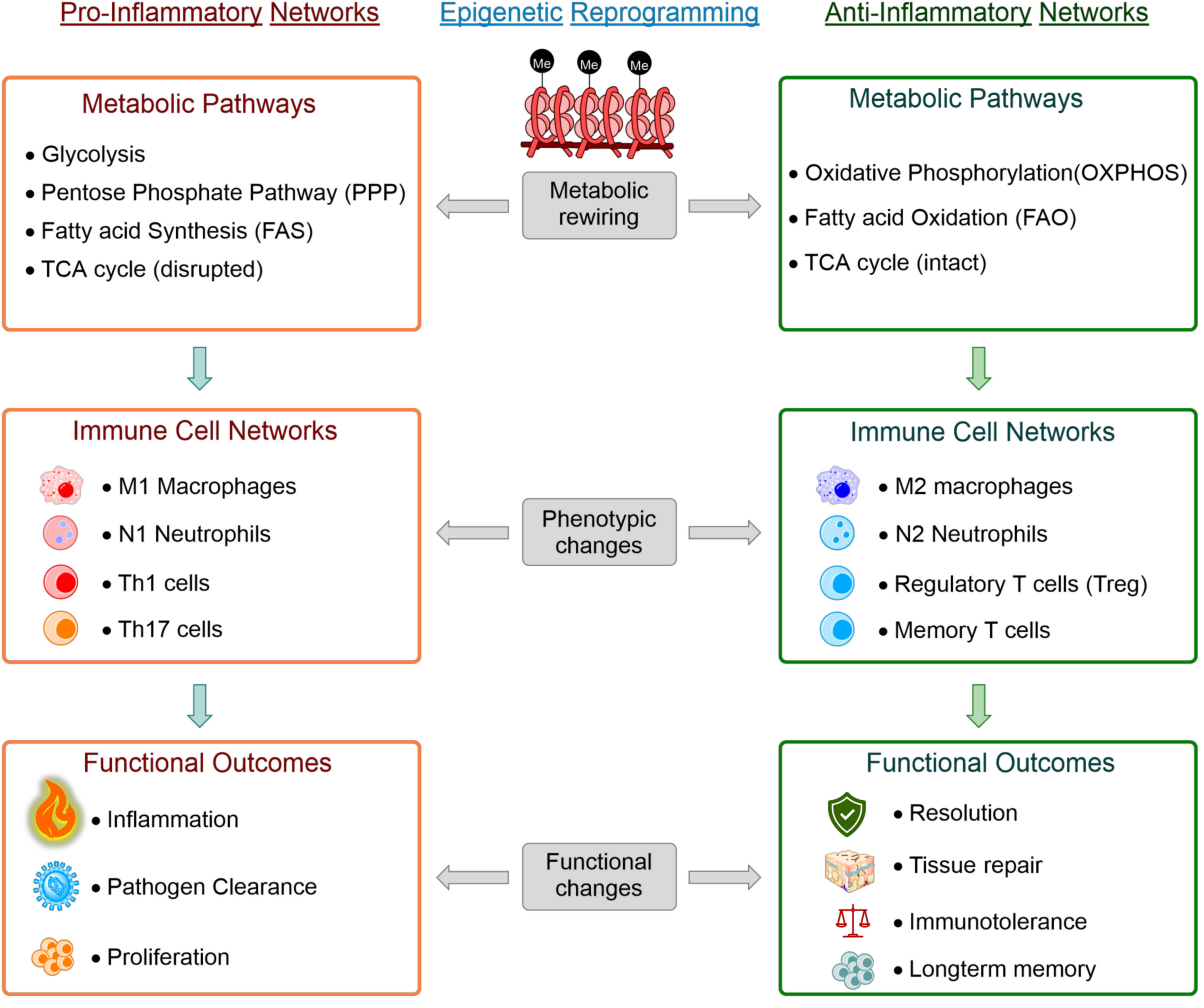
Immunometabolic and epigenetic regulation of immune cell plasticity. Reconfiguration of metabolic pathways in innate and adaptive immune cells induces phenotypic and functional adaptations that establish a dynamic equilibrium between pro-inflammatory and anti-inflammatory immune networks required for maintenance of immunological homeostasis.

Immunometabolic reprogramming refers to the dynamic reconfiguration of metabolic pathways in innate and adaptive immune cells, which induce epigenetic, phenotypic, and functional changes that shape immune cell plasticity and inflammatory outcomes, as illustrated in **Figure 3**. In the context of trained immunity and metaflammation, macrophages have emerged as one of the most extensively studied models of immunometabolic plasticity (59–62). While *in vivo* macrophages exhibit transcriptionally dynamic behavior shaped by the tissue microenvironment, *in vitro* they can be broadly classified into two functional subgroups: classically activated M1 macrophages, activated by Th1 cytokines such as TNF-*α* and IFN-*γ* to induce pro-inflammatory cytokine responses; and alternatively activated M2 macrophages, activated by Th2 cytokines such as IL-4 and IL-13 to mediate resolution of inflammation and tissue repair through antiinflammatory responses (59–61, 63). In resting states and during M2-mediated inflammation resolution, energy demands are low and cells rely on thermodynamically efficient metabolic pathways — oxidative phosphorylation (OXPHOS) and fatty acid oxidation (FAO) in conjunction with an intact tricarboxylic acid (TCA) cycle (60, 63). Upon activation or phenotypic switching from M2 to M1, however, cells must rapidly access substrates to power effector functions including phagocytosis, inflammatory cytokine production, and antigen presentation. This involves a shift toward aerobic glycolysis, glutaminolysis, pentose phosphate pathway (PPP) and fatty acid synthesis (FAS), accompanied by disruption of the TCA cycle — fulfilling the high energetic and biosynthetic requirements of the activated inflammatory state (60, 63).

Metabolic disorders characterized by metaflammation — including T2D and obesity — involve functional reprogramming of macrophages from the M2 to the M1 phenotype. In obese AT, M1-like macrophages accumulate due to increased amounts of free fatty acids, cholesterol, and LPS, driving AT inflammation (59). Adipocyte hypertrophy in AT leads to reduced tissue vascularization and hypoxia, which activates transcription factors such as hypoxia inducible factor (HIF-1*α*) and NF-*κ*B (36). This induces activation of inflammatory kinases such as c-jun N-terminal kinase (JNK) and inhibitor of *κ* kinase (IKK), thereby enhancing expression of pro-inflammatory genes such as IL-6, TNF-*α*, IFN-*γ*, and IL-1*β*. This promotes infiltration of M1-like macrophages to AT, accentuating the pro-inflammatory environment and inhibiting insulin signaling (36). Immunometabolic reprogramming has also been extensively characterized in trained immunity. Training of monocytes with BCG resulted in increased H3K4me3 and decreased H3K9me3 at the promoter regions of TNF-*α*, IL-6, and metabolic regulators including mammalian target of rapamycin (mTOR), hexokinase-2 (HK2), and phosphofructokinase (PFKP), with concomitant increased mRNA expression of these enzymes (62). Moreover, human monocytes trained with *β*-glucan show increased expression of glycolytic enzymes including HK2 and PFKP, resulting in enhanced glucose consumption, lactate production, and an altered NAD^+^/NADH ratio — a Warburg-like metabolic shift (62, 64). This metabolic reprogramming prepares cells for rapid pro-inflammatory cytokine responses and is accompanied by changes in histone marks including H3K4me1, H3K4me3, and H3K27ac at promoter regions of inflammatory genes (65).

Beyond monocytes and macrophages, adaptive T lymphocytes also exhibit distinct metabolic processes based on their functionality. Pro-inflammatory Th1 and Th17 cells rely largely on glycolysis and glutaminolysis to support rapid proliferation and cytokine production, whereas regulatory T cells (Tregs) preferentially utilize OXPHOS and FAO (66, 67). Moreover, B cells also undergo substantial metabolic remodeling during activation and differentiation into antibody-producing plasma cells (68, 69). Activation and differentiation of B cells are accompanied by increased OXPHOS, mitochondrial remodeling, TCA cycle activity, and nucleotide biosynthesis, whereas later stages of plasma-cell differentiation impose additional metabolic demands required for sustained antibody production and immune memory.

Lipid metabolism has recently emerged as a major regulator of plasticity of immune cells. FAO generally supports anti-inflammatory and tissue-repair immune cell phenotypes, whereas increased FAS and dysregulated lipid storage are typically associated with inflammatory activation (67, 70, 71). FAO and FAS play contrasting roles in adaptive immunity: FAO promotes the generation of Tregs and inhibits effector T cell polarization, while FAS supports the proliferation and function of pro-inflammatory effector T cells. FAO also promotes long-lived memory CD8+ T cells, which are required for sustained immune function. Cholesterol metabolism further contributes to inflammatory signaling through effects on membrane organization, macrophage activation, and foam-cell formation (72). The differential utilization of FAO and FAS in immune cells underscores the central role of lipid metabolism in shaping inflammatory outcomes. This is particularly important in the tumor microenvironment (TME), where metabolic competition and lipid-rich conditions drive distinct immunometabolic adaptations in both adaptive and innate immune cells.

#### 2.4.1 Metabolic plasticity in the tumor microenvironment: implications for inflammaging

The TME constitutes a metabolically competitive ecosystem composed of tumor cells, stromal cells, immune cells, and cancer-associated fibroblasts. The high energetic demands of rapidly proliferating tumor cells drive a metabolic switch from OXPHOS to aerobic glycolysis, resulting in nutrient deprivation, hypoxia, and lactate accumulation, thereby establishing an immunosuppressive milieu that profoundly reshapes immune cell metabolism and functionality (70, 73–75). This metabolic stress profoundly impacts the functionality of T cells, leading to functional impairment and exhaustion of CD8+ cytotoxic T cells. Moreover, the lipid-rich environment promotes differentiation of CD4+ T cells into immunosuppressive Tregs. Although Tregs primarily rely on the TCA cycle and OXPHOS, metabolic stress shifts their reliance toward FAO, conferring survival advantages in the glucose-deficient, lactate-rich microenvironment (70, 71).

Metabolic reprogramming also occurs in tumor-infiltrating myeloid cells, including tumor-associated macrophages (TAMs) and tumor-associated neutrophils (TANs). The TME reconfigures metabolic processes in TAMs, promoting their transition from an anti-tumor M1-like phenotype to a pro-tumor and immunosuppressive M2-like phenotype (70, 71). Recent single-cell RNA sequencing studies in colorectal cancer have identified a distinct immunosuppressive TAM subset, the SPP1+ phenotype, which exhibits increased glucose metabolism and FAO, while retaining inflammatory features through production of pro-inflammatory cytokines including IL-1*β* and TNF-*α* (76, 77). These findings highlight that immunometabolic reprogramming in the TME generates a spectrum of macrophage states, extending beyond the traditional M1/M2 dichotomy. Similar to TAMs, TANs also exhibit significant plasticity and can adopt anti-tumor N1 or pro-tumor N2 states, governed by the balance between IFN-*β* and TGF-*β* signaling (70, 74, 75). N1 TANs, associated with pro-inflammatory activity, rely on aerobic glycolysis, whereas in the hypoxic, nutrient-deprived TME, neutrophils adopt an immunosuppressive N2 phenotype, relying on anaerobic glycolysis, OXPHOS, and FAO (70, 75). These phenotypic shifts are driven by HIF-1*α*, NF-*κ*B, and mTOR signaling pathways, activated in response to the metabolic stress in the TME (70, 75). Interestingly, beyond the TME, neutrophils exhibit enhanced anaerobic glycolysis also in chronic inflammatory disorders as they encounter hypoxic and inflammatory microenvironments in inflamed tissues, where similar metabolic adaptations are regulated by HIF-1*α* and NF-*κ*B (75). Notably, HIF-1*α* has also been found to be an important regulator of trained immunity, promoting mTOR-mediated aerobic glycolysis, enhancing responsiveness to secondary stimulation (64).

Given the shared metabolic and inflammatory mechanisms between the TME and inflamed tissues, insights from TME-mediated immunometabolic reprogramming may provide a useful framework for understanding inflammaging and interindividual variability in susceptibility to inflammatory disorders. Beyond the interplay between epigenetic and immunometabolic mechanisms discussed in this section, genetic polymorphisms in glycolytic enzymes HK2 and PFKP also contribute to interindividual variability in trained immunity (62). Taken together, it can be hypothesized that interindividual variability in epigenetic and immunometabolic reprogramming, together with genetic polymorphisms in enzymes involved in metabolism and inflammation, may explain differences in inflammaging susceptibility. Thus, quantifying this variability involves developing biomarkers that capture both the genetic and epigenetic components of metabolic-inflammation axes.

### 2.5 Biomarkers of inflammaging

Biomarkers of inflammaging can be broadly divided into two categories: molecular biomarkers that measure circulating levels of specific inflammatory mediators such as pro-inflammatory cytokines discussed above, and functional or mechanistic biomarkers that measure genetic or epigenetic components of inflammaging. Unlike the former biomarkers, the latter are largely independent of temporary changes in diet and lifestyle factors over a short span of time and constitute more robust measures of inflammaging. In the following, we will discuss one such class of biomarkers of biological aging called epigenetic clocks.

#### 2.5.1 Epigenetic clocks

Individuals of the same chronological age can vary markedly in their biological aging trajectory and disease susceptibility. Biological age estimated from epigenetic markers captures this variation (78). When biological age exceeds chronological age, it is termed epigenetic age acceleration ( $e_{\text{AA}}$) (78), calculated using Equation 1 as:

(1) $$e_{\text{AA}}=\text{DNAmAge}-\text{ChronologicalAge},$$

where DNAmAge is the biological age estimated from DNA methylation patterns using a trained algorithm. Individuals with positive *e*_AA_ age faster biologically than their calendar years suggest, while those with negative *e*_AA_ age more slowly. The first epigenetic clock was developed by Horvath (78), using 353 CpG sites across multiple tissues and cell types, demonstrating that cancer is associated with epigenetic age acceleration (78). The Hannum clock (79), developed from genome-wide methylation profiling of whole blood, introduced the concept of apparent methylomic aging rate (AMAR) and demonstrated that males exhibit higher AMAR than females. Second-generation clocks, such as PhenoAge (80) and GrimAge (81), moved beyond age estimation to predict morbidity and mortality. PhenoAge (80) was trained on a phenotypic age score derived from 10 clinical markers including chronological age, albumin, creatinine, glucose, and C-reactive protein (CRP). Third-generation clocks, such as the DunedinPACE clock, shifted from measuring the current state of biological aging to measuring its pace (82).

Principal component-based (PC-based) epigenetic clocks represent a methodological advancement that addresses the overfitting limitations of earlier clocks by compressing methylation data into biologically interpretable axes of aging (83, 84). PCAge, its streamlined variant LinAge, and the CALERIE-adapted CALinAge integrate high-dimensional clinical data into a reduced feature space where distinct principal components reflect coordinated physiological processes including inflammation, metabolic dysregulation, and organ system decline (84). Moreover, PC-based versions of second-generation clocks — PCGrimAge and PCPhenoAge — demonstrate improved predictive performance for mortality and functional decline compared to their original counterparts, while retaining biological interpretability (85). By reducing high-dimensional epigenetic data into interpretable PCs, such clocks provide a lower dimensional systemic observable, which is analogous to mapping multi-dimensional genotypic variation into a three-dimensional phenotypic variation (Prakriti) through ayurgenomics studies (14, 86, 87).

## 3 Prakriti in Ayurveda

In Ayurveda, Prakriti refers to the unique constitutional phenotype of an individual, assessed through physical, physiological, mental, and emotional characteristics, manifested as Vata, Pitta, and Kapha doshas. It represents an individual’s unique state of homeostasis, established at conception based on the relative proportions of these doshas in the mother’s womb. Each dosha governs distinct biological functions through its associated phenotypic attributes called Gurvadi Gunas (88). Vata governs physiological motion, cellular turnover, and catabolic processes, and is associated with an efficient agile responsive system (laghu), increased motility (chala), reduced tissue hydration (rooksha), causative of structural irregularities (khara), and thermolytic effects (sheeta). Pitta regulates digestion, assimilation, and excretion, and is associated with Gurvadi Gunas such as ushna, tikshna, amla, sara, and katu. Kapha governs tissue formation, nourishment, and repair, owing to its association with Gurvadi Gunas such as snigdha, manda, sthira, mrudhu, and sheeta. Seven Prakriti types are possible based on the number of predominant doshas: Vata, Pitta, Kapha; dwandwaja (dual) types of Vata-Pitta, Pitta-Kapha, Vata-Kapha; and the Sama Prakriti with equal proportions of doshas. Any deviation from the homeostatic state of an individual’s Prakriti may lead to disease, a condition referred to as Vikriti, which denotes an imbalance or altered state of the Doshas (89).

Ayurgenomics studies have provided empirical support for the Ayurvedic concept of Prakriti by demonstrating systematic genotypic differences between Prakriti types. A landmark study by Prasher et al. identified 251 genes differentially expressed between extreme prakriti groups, encompassing pathways related to cell division, metabolism, and immunity (13). A genome-wide SNP analysis by Govindaraj et al. identified differences in 52 SNPs between Prakritis, and also found that the SNP rs11208257 in phosphoglucomutase-1 (PGM1) gene correlates with phenotype of Pitta (14), which leads to increased glucose consumption and impairment of glycogen pathway, which might have implications for T2D (90). While an individual’s physiology involves thousands of biological variables, Ayurvedic tradition, through centuries of experiential medical practice, appears to have identified the three most significant axes of variation—Vata, Pitta, and Kapha—to explain the majority of phenotypic diversity, as corroborated by unsupervised and supervised machine learning algorithms in modern ayurgenomics studies (14, 91, 92). A detailed discussion of how evidence from ayurgenomics studies help distinguish different Prakritis in terms of variations in metabolism and inflammatory responses is provided in Section 4.1.

### 3.1 Assessment of Prakriti

Traditional Prakriti assessment is qualitative, relying on a detailed interaction between patient and Ayurvedic physician encompassing morphological features, such as skin complexion, hair texture, body proportions; metabolic characteristics including digestive quality, appetite, bowel habits; psychological attributes such as mental endurance, memory, sleep quality, as well as analyzing behavioral patterns. Such an interaction helps clinician understand the immunological history of the individual — a practice that finds resonance in the modern concept of immunobiography. Efforts to develop standardized, objective assessment tools have produced instruments such as the Prototype Prakriti Analysis Tool (PPAT) (93) and AYUSOFT (94). Objective parameters under investigation include anthropometric indices, skin and hair phenotyping, spirometry for vital capacity, heart rate variability (HRV) for autonomic function, electrogustometry for taste threshold, gut microbiome stratification, and body temperature profiling (91). A recent study analyzed challenges in Prakriti assessment using 64 distinct Prakriti Assessment Tools (95), of which 20 underwent formal validation in terms of accuracy and reliability. Among these, the CCRAS Prakriti Assessment Software (CCRAS-PAS) and the AyuSoft-based Constitutional Prakriti Indicator (ACPI) exhibited the strongest methodological rigor. Challenges remain regarding test-retest reliability, dimensionality assessment, and cross-population validation.

### 3.2 Ayurvedic perspective on aging

Ayurveda conceptualizes aging through the term Jara or Vayo, derived from the Sanskrit root “Jiryati Iti Jara” — that which decays is aging. The body (Sharira) is itself defined as “Shiryati Iti Shariram” — that which is subject to decay. Aging is thus understood not as an aberration but as the natural, inevitable catabolic trajectory of the body, culminating in the progressive degeneration of tissues and decline in physiological function. Charaka Samhita, Sloka 122 of Vimana Sthana (96), categorizes life span (Ayu) into three distinct phases of Vayah (biological age):

1. *Balam: Sleshma Dhatu Praayam* – childhood and young age (0–30 years) that involves tendency for increase in Kapha dosha.
2. *Madhyam: Pitta Dhatu Praayam* – middle age (31–60 years) that involves tendency for increase in Pitta dosha.
3. *Jirnam: Vayu Dhatu Praayam* – old age (61–100 years) that involves tendency for increase in Vata dosha.

Interestingly, the Sloka further states that the three-phase division should be interpreted relative to the individual’s expected Ayu (lifespan), which varies between Prakriti — meaning that the absolute age ranges must be scaled based on constitutional Ayu.

Ayurveda estimates individual life span through a comprehensive assessment across nine domains (88), each rated as Shrestha or Pravara (best, score 3), Madhyama (moderate, score 2), or Avara (worst, score 1): 1. Prakriti; 2. Sara: a qualitative assessment of tissue integrity and functional excellence across the major body tissues, including blood, integumentary structures (skin), muscle, adipose tissue, bone, bone marrow, and reproductive tissues; 3. Samhanana: reflecting structural robustness and musculoskeletal integrity; 4. Pramana: the assessment of body proportions and anthropometric characteristics; 5. Satmya: denoting physiological adaptability and dietary compatibility; 6. Sattva: representing psychological resilience and cognitive-emotional stability; 7. Aharashakti: indicating digestive efficiency and metabolic capacity; 8. Vyayamashakti: reflecting exercise tolerance and physical endurance; and 9. Vayah Pariksha: the assessment of functional or biological age. The composite score across these nine dimensions determines whether an individual’s lifespan falls in the Pravara, Madhyama, or Avara range (88).

Critically, Prakriti is the first and arguably most fundamental of these nine determinants. Charaka Samhita explicitly associates Prakriti type with Ayu: Kapha or Sama individuals are classified as exceptional (Pravara/Uttama/Shrestha), with a high life expectancy attributable to high strength (Bala) and robust immune responses (Ojas) (88). Pitta individuals are classified as Madhyama with moderate life span owing to moderate strength and inflammatory immune responses. Vata individuals are classified as Avara (Hina) with relatively lower life span owing to lower innate strength and weak immune capacity. The framework for Prakriti-based Ayu expectancy, as well as the aforementioned three-phase division of Vayah based on the estimated Ayu, provides the conceptual foundation for our hypothesis connecting Prakriti with differential susceptibility to inflammaging.

## 4 Hypotheses connecting inflammaging and Prakriti

As discussed in the Introduction, inflammaging is characterized by the progressive accumulation of inflammatory load over time, eventually reaching a threshold beyond which susceptibility to chronic age-related diseases increases, manifesting as accelerated aging. Within the two-hit theory (4), inflammaging arises from the interaction of two complementary components: (i) a baseline inflammatory tone determined by the genetic makeup of the individual (*B*); (ii) an epigenetically driven component (*E*) shaped by environmental and lifestyle factors. The first component corresponds to genetic predisposition, reflecting the presence or absence of robust gene variants that determine an individual’s inherent inflammatory baseline. It can be assumed that the inflammatory baseline, within the Ayurvedic framework, is determined by Prakriti, the relative proportions of the three doshas at conception, which constitutes the homeostasis of an individual. Differences in Prakriti are therefore expected to reflect differences in genetic inflammatory tone. Since Prakriti corresponds to the genetically fixed baseline component *B*, its evaluation requires instruments that directly capture germline genetic architecture, independent of epigenetic influence. We therefore propose the GII — a composite germline measure constructed from SNPs in genes encoding a few representative pro-inflammatory and anti-inflammatory cytokines — as a quantitative measure of genetic inflammatory tone. The GII and its constituent SNP panel are described in detail in Section 4.3.

The second component, *E*, is the epigenetic factor encompassing all sources of deviation from the constitutional baseline — both extrinsic environmental and lifestyle exposures and intrinsic physiological dysregulation — which collectively accumulate over the lifespan through epigenetic and immunometabolic reprogramming. In Ayurveda, this component corresponds to Vikriti, the dynamic deviation from constitutional homeostasis. Isolating the epigenetic component from the genetic component might be challenging owing to the dynamic nature of the former. Instead, we capture the cumulative effect of both by considering *e*_AA_ as the primary observable. Importantly, *e*_AA_ cannot be used to isolate *B* independently, necessitating the use of aforementioned GII measure to capture the genetic baseline component. Within the proposed framework, *e*_AA_ can be expressed using Equation 2 as:

(2) $$e_{\text{AA}}=g(\text{B})+\int_{0}^{t}E(\tau )d\tau \approx f(\text{GII})+\int_{0}^{t}E(\tau )d\tau ,$$

where the integral term corresponds to the cumulative epigenetic inflammatory load accumulated through all sources of Vikriti over the lifespan, while *f*(GII) constitutes an approximation to the baseline inflammatory component g(B).

### 4.1 Prakriti as a unique constitutional immunometabolic phenotype: hypothesis-I

In this section, we construct our hypotheses on a potential association between inflammaging susceptibility and Prakriti by integrating preliminary evidence from ayurgenomics studies and Ayurvedic clinical observations with constitutional concepts described in classical Ayurvedic texts. This evidence-informed reinterpretation of Ayurvedic concepts provides a basis for proposing experimentally testable and falsifiable predictions within the framework of inflammaging.

#### 4.1.1 Metabolic manifestations of Prakriti

Classical Ayurvedic texts describe each Prakriti through a characteristic and constitutionally fixed metabolic phenotype (88, 97). Pitta Prakriti is characterized by Tikshnagni — a strong, rapid, and intense metabolic phenotype — governing digestion, assimilation, and energy transformation. Kapha Prakriti is characterized by Mandagni — a slow and efficient metabolic phenotype — governing tissue formation, structural integrity, and nourishment. Vata Prakriti is characterized by Hinagni or Vishamagni — an irregular and variable metabolic phenotype — reflecting its Yogavahi (plasticity) nature. Specifically, Pitta metabolic phenotype is supported by emerging ayurgenomics evidence: a genome-wide SNP analysis by Govindaraj et al. identified PGM1, a key enzyme in glycolysis and related metabolic pathways, as significantly associated with Pitta Prakriti across diverse Indian populations (14). Ghodke et al. studied the association between CYP2C19 gene polymorphisms and differences in Prakriti (98). Specifically, they reported that an extensive metabolizer genotype is predominant in Pitta Prakriti individuals, while a poor metabolizer genotype is more prevalent in Kapha Prakriti. Based on these observations, the authors proposed that Pitta and Kapha individuals may require relatively higher and lower doses, respectively, of CYP2C19 substrate drugs such as diazepam, omeprazole, and proguanil. Beyond genes directly associated with metabolism, ayurgenomics studies have also identified differential expression and allele frequency variation in other regulatory genes such as the EGLN1 gene (99), an oxygen sensor gene that *negatively* regulates the activity of HIF-1*α* stability under normoxic conditions. Specifically, the rs479200 allele of EGLN1, associated with higher gene expression, was reported at significantly higher frequency in Kapha Prakriti individuals compared to Pitta Prakriti (99). Higher expression of EGLN1 in Kapha may therefore limit baseline HIF-1*α* activation, potentially increasing the threshold for innate immune activation. In contrast, Pitta individuals with lower baseline may possess a greater propensity to maintain an alert innate immune system, through enhanced HIF-1*α*/mTOR signaling in trained immunity (64).

#### 4.1.2 Inflammatory manifestations of Prakriti

Classical Ayurvedic texts describe constitutional differences in immune strength across Prakriti types. Vata Prakriti individuals are characterized by Alpa Bala and Alpa Ojas — low vital essence and low immune strength — rendering them more vulnerable to disease. Kapha Prakriti individuals, in contrast, are described as having Uttama Bala and Ojas, i.e., superior intrinsic strength and robust immune responses (88). Interestingly, an immunophenotyping study of 222 healthy Prakriti-stratified individuals found that Kapha individuals exhibited significantly higher expression of CD25 (activated B cells) and CD56 (natural killer cells) compared to Vata individuals, indicating constitutionally stronger baseline immune responses in Kapha and weaker innate and adaptive immune readiness in Vata (100). Conversely, Pitta individuals showed higher expression of CD14 (monocytes), suggesting a greater capacity for initiating acute innate immune responses.

Conventionally, Ayurveda associates aggravated Vata with instability in physiological regulation, heightened responsiveness to internal and external stressors, and disturbances in neural, gastrointestinal, and systemic functions. Based on these characteristics, it was proposed that the vagus nerve may serve as a potential biomarker for specific functions of Vata dosha, as both are involved in maintaining neural, respiratory, digestive, and gut–brain homeostasis (101). Emerging evidence from integrative physiology suggests that these features may correspond to alterations in autonomic nervous system regulation, particularly vagal activity. Specifically, a study examining heart rate variability (HRV), a primary non-invasive measure of vagal tone, has demonstrated differential autonomic responses among Prakriti types, with Vata-predominant individuals exhibiting significantly greater reductions in parasympathetic activity during orthostatic stress compared to Kapha and Pitta types (102). Such exaggerated vagal withdrawal in Vata individuals suggests a labile vagal brake that disengages more readily under physiological challenge. Mechanistically, the vagus nerve plays a central role in the cholinergic antiinflammatory pathway, through which vagal signalling modulates immune activation and suppresses excessive inflammatory responses (103). Consequently, diminished vagal tone associated with Vata dysregulation may contribute to autonomic imbalance and impaired inflammatory control. Taken together, these empirical observations and classical Ayurvedic descriptions of Vata Prakriti suggest that *Vata dosha constitutes a key regulator of inflammation*, analogous to the functionality of vagal nerve, and vitiation of Vata dosha leads to inflammatory imbalance.

Based on all distinct empirical observations on differences in metabolism and inflammatory immune responses presented above, one expects Vata individuals to be more susceptible to inflammaging due to intrinsic tendency for Vata dosha aggravation, while Kapha individuals are expected to be least susceptible owing to robust adaptive immune responses. On the other hand, Pitta individuals are expected to exhibit an alert or trained innate immune system. These inferences lead naturally to our first hypothesis:

**Hypothesis-I**. Different Prakriti types exhibit systematic differences in inflammatory genotypes. Baseline inflammatory tone can be quantitatively captured using the Genetic Inflammatory Index (GII), with Vata and Pitta Prakritis expected to exhibit high pro-inflammatory scores (GII *>* 0), while Kapha is expected to exhibit high anti-inflammatory score (GII *<* 0).

### 4.2 Vikriti and inflammaging: hypothesis-II

While Hypothesis-I posits that Prakriti determines baseline genetic susceptibility to inflammaging, the epigenetic contribution to inflammaging can be interpreted in terms of Vikriti – dosha imbalance from constitutional homeostasis – owing to cumulative exposure to inflammatory stimuli such as pathogens, diet, and lifestyle over time. A mapping between dosha imbalance over the life span and inflammatory imbalance observed in inflammaging finds support from a classical verse in Ashtanga Hridaya (97):

*Vayo ahoratri bhuktanam te antah madhyadigah kramath.*

The sloka states that across the lifespan (Vayo), day (Aho) and night (Ratri), and digestion (Bhukthanam), the three doshas dominate in their canonical sequential order (Kramath): Vata at the end phase (Antah), Pitta in the middle phase (Madhya), and Kapha at the beginning phase (Adigah). Applied to the life span dimension (Vayo), the following interpretation emerges (91): Kapha dosha aggravation is typical in childhood and youth reflecting the anabolic and anti-inflammatory immunological state; Pitta dosha aggravation is typical in middle age reflecting the peak metabolic-inflammatory activity that initiates the accumulation of inflammatory load; Vata dosha aggravation is common in old age manifesting the catabolic, dysregulated, chronic pro-inflammatory state that constitutes inflammaging. The age-progressive dosha predominance described in the Ashtanga Hridaya verse bears resemblance to the age-related inflammatory imbalance, established in inflammaging research.

The hypothesized regulatory role of Vata dosha in inflammation is further supported by clinical evidence in chronic inflammatory conditions, such as rheumatoid arthritis (RA). Specifically, a case-control study demonstrated that Vata Prakriti is significantly associated with RA susceptibility, with an odds ratio of 23.9, indicating that Vata individuals are far more likely to develop the disease compared to Pitta and Kapha individuals (104). Corroborating this finding, genetic studies have shown that Vata individuals with RA exhibit an inflammatory gene signature involving IL-1*β*, TNF-*α*, and CD40, whereas Pitta-predominant patients show oxidative stress pathway associations (105), indicating that once disease is established, the underlying inflammatory pathways differ by Prakriti in a manner consistent with the constitutional baseline differences documented in Hypothesis-I. Beyond the genetic level differences, Rotti et al. demonstrated that the three Prakriti types are distinguished by genome-wide DNA methylation signatures, with specific markers such as SOX11 and LHX1 serving as epigenetic anchors for Pitta and Vata respectively (106). This suggests that individuals do not start from a uniform epigenetic zero-point; rather, the constitutional DNA methylation landscape of each Prakriti may predetermine the sensitivity and baseline rate of epigenetic age acceleration. By choosing age-matched cohorts from a broadly comparable inflammatory stressor exposure, disease history, and lifestyle, residual differences in epigenetic age acceleration across Prakriti groups will reflect differences in the combined constitutional baseline. This leads to our second hypothesis:

**Hypothesis-II**. Within age-matched cohorts under broadly comparable environmental and lifestyle conditions, Prakriti is expected to significantly influence biological aging as measured by *e*_AA_. Specifically, individuals of Vata Prakriti are hypothesized to exhibit higher baseline inflammatory tone and correspondingly higher *e*_AA_, followed by Pitta, while Kapha and Sama Prakriti individuals are expected to exhibit lower *e*_AA_ values, following the ordering in Equation 3:

(3) $$e_{\text{AA}}(\text{Vata})>e_{\text{AA}}(\text{Pitta})>e_{\text{AA}}(\text{Kapha})>e_{\text{AA}}(\text{Sama}).$$

**Figure 4:**
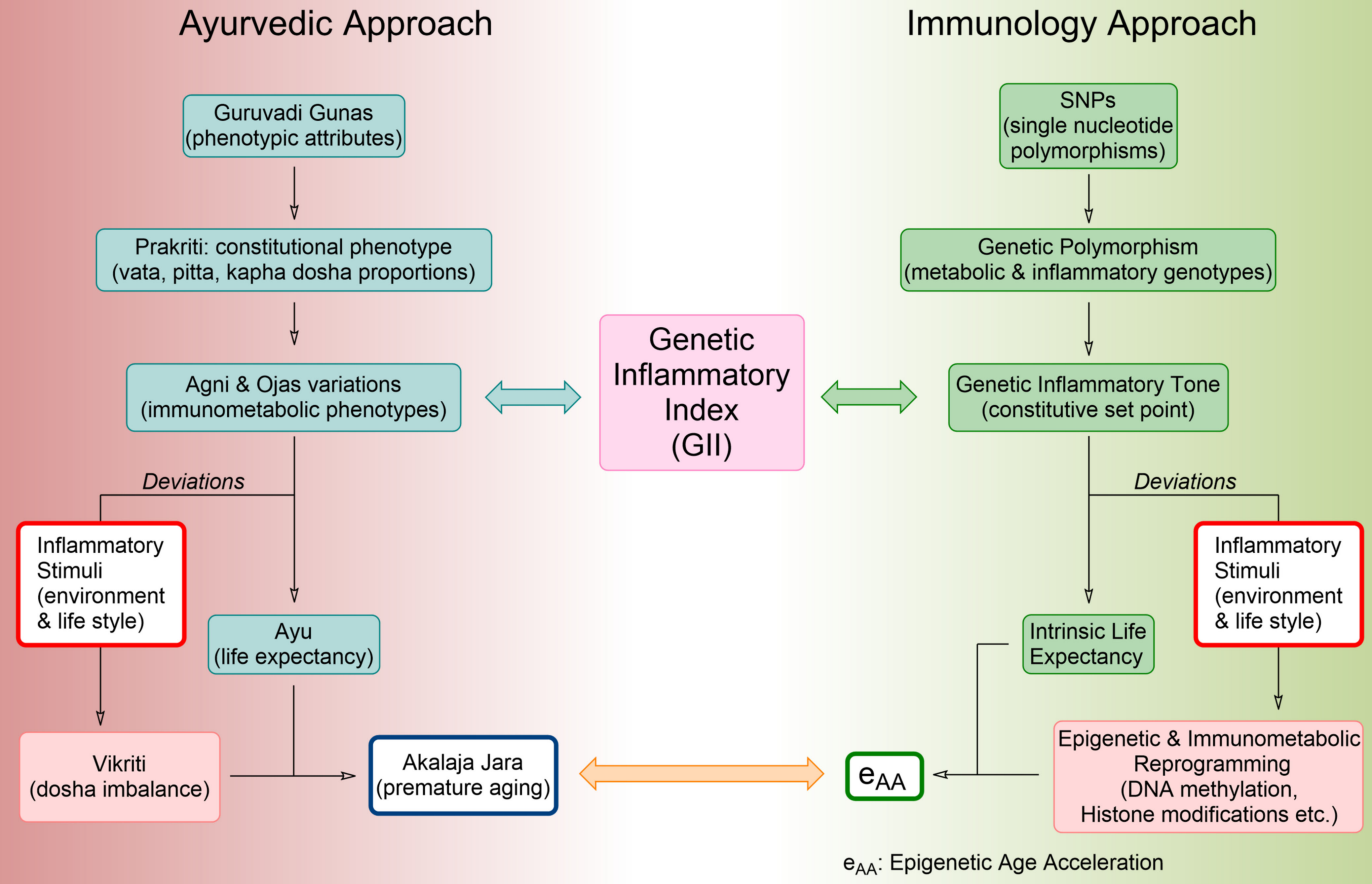
Conceptual framework integrating Ayurvedic and Immunology approaches to understand interindividual variations in inflammaging. Left: Ayurvedic approach maps constitutional phenotypic attributes (Guruvadi Gunas) to Prakriti, a unique constitutional phenotype that represents relative proportions of Vata, Pitta, and Kapha doshas of an individual established at conception. Variations in Prakriti types manifest as differences in Agni (metabolism) and Ojas (immune responses), which can be quantified using the proposed Genetic Inflammatory Index (GII). Prakriti is a critical factor in determining baseline Ayu (life expectancy). However, inflammatory stimuli drive Vikriti (dosha imbalance), which can lead to Akalaja Jara (premature aging). Right: Immunology approach maps single nucleotide polymorphisms (SNPs) in genes related to metabolism and inflammation to a constitutive genetic baseline inflammatory tone, quantified through GII. Inflammatory stimuli drive epigenetic and immunometabolic reprogramming, which constitute an epigenetic load on the baseline genetic component. The *cumulative* effect of both the components determines epigenetic age acceleration (*e*_AA_), which integrates both approaches under the proposed hypotheses.

A unified schematic representation of both hypotheses is presented in **Figure 4**, which highlights the convergence of Ayurvedic constitutional stratification approach and contemporary immunology approach through the observables GII and *e*_AA_, which constitute the measurables of Hypothesis-I and Hypothesis-II, respectively.

### 4.3 Hypotheses testing

The hypotheses presented above generate experimentally testable predictions linking Prakriti to baseline genetic inflammatory tone, and sensitivity to epigenetic changes. The genetic component can be quantified using the GII framework by Prakriti-stratified cohort studies as explained below.

#### 4.3.1 Cohort design and Ayurvedic Prakriti assessment

We propose a Prakriti-stratified cohort design based on two key dimensions: chronological age and Prakriti type. Individuals are first stratified into non-overlapping age cohorts – young (15–25 years), middleaged (35–45 years), and older (55–65 years) – ensuring that comparisons across Prakriti groups are not confounded by chronological age. This stratification is also consistent with classical Ayurvedic descriptions of age-dependent dosha predominance (82). Within each age cohort, individuals are classified into Vata, Pitta, and Kapha Prakriti types using standardized Ayurvedic assessment tools such as the Prototype Prakriti Analysis Tool (PPAT) (93) and AyuSoft (94), administered by trained Ayurvedic physicians. To ensure robustness, classification is based on constitutionally stable traits and requires concordance between physician assessment and software-based evaluation, with a minimum dominance threshold (e.g., ≥ 60% single dosha dominance). This Ayurvedic assessment generates a Prakriti vector, given by Equation 4 that captures relative proportions of Vata, Pitta, and Kapha doshas, and we hereafter refer it to as the Ayurvedic Prakriti vector ${\vec{P}}_{A}\in {\mathbb{R}}^{3}$ as:

(4) $${\vec{P}}_{A}=[\begin{array}{c} V\\ P\\ K \end{array}],$$

and its normalized version is obtained using Equation 5 as:

(5) $${\hat{P}}_{A}=\frac{{\vec{P}}_{A}}{|{\vec{P}}_{A}|}=\frac{{\vec{P}}_{A}}{\sqrt{V^{2}+P^{2}+K^{2}}}.$$

#### 4.3.2 Construction of genetic Prakriti vector

To quantify the baseline genetic inflammatory tone, we construct the genetic Prakriti vector ${\vec{P}}_{G}\in {\mathbb{R}}^{3}$ from a composite germline score derived from SNPs in genes encoding key pro-inflammatory, anti-inflammatory, and pleiotropic cytokines. It captures the constitutional inflammatory architecture of the individual, and within our hypothesis corresponds to the three dosha components using Equation 6 as:

(6) $${\vec{P}}_{G}=[\begin{array}{c} V_{s}\\ P_{s}\\ K_{s} \end{array}],$$

and its normalized version given by Equation 7 as

(7) $${\hat{P}}_{G}=\frac{{\vec{P}}_{G}}{|{\vec{P}}_{G}|}=\frac{{\vec{P}}_{G}}{\sqrt{V_{s}^{2}+P_{s}^{2}+K_{s}^{2}}}.$$

Here, $V_{s}$, $P_{s}$, and $K_{s}$, are normalized dosha component scores computed using Equation 8 as:


(8) $$V_{s}=\sum_{i=1}^{n_{V}}\frac{D_{i}}{2n_{V}},\;P_{s}=\sum_{j=1}^{n_{P}}\frac{D_{j}}{2n_{P}},\;K_{s}=\sum_{k=1}^{n_{K}}\frac{D_{k}}{2n_{K}}.$$

Here, *n_V_*, *n_P_*, and *n_K_* denote the number of SNPs in the Vata, Pitta, and Kapha groups, respectively. The *D_i_*, *D_j_*, and *D_k_* represent inflammatory cytokine allele dosage scoring, which assigns 0 points for homozygous non-target genotype, 1 point for heterozygous, and 2 points for homozygous target, where the denominator represents the maximum possible score for each component. Within the proposed Hypothesis-I, a natural correspondence is expected between the genetic Prakriti vector ${\vec{P}}_{G}$ and the Ayurvedic Prakriti vector ${\vec{P}}_{A}$: a profile with the highest *V_s_* is expected to correlate with Vata Prakriti; the highest *P_s_* to Pitta Prakriti; the highest *K_s_* to Kapha Prakriti; and balanced scores correspond to Sama Prakriti.

The selection of cytokine SNPs included in calculating ${\vec{P}}_{G}$ is based on previous studies demonstrating their functional impact on cytokine expression and inflammatory tone. Promoter polymorphisms in key cytokines such as IL-6 (rs1800795) (107) and TNF-*α* (rs1800629) (108) have been shown to modulate transcriptional activity and circulating cytokine levels, thereby influencing the intensity of pro-inflammatory responses. Similarly, variants in IL-17A (rs2275913) (109) and MCP-1/CCL2 (rs1024611) (110) are associated with enhanced pro-inflammatory signaling and susceptibility to chronic inflammatory conditions. Polymorphisms in TNF-*α* and IL-1*β* are among the most extensively studied genetic determinants of acute inflammatory intensity, and IFN-*γ* variants regulate Th1-mediated immune responses (111), characteristic of Pitta-type acute systemic inflammation. It is notable that cytokine classification within this framework is based on dominant functional roles in specific inflammatory contexts rather than strict exclusivity. Certain cytokines exhibit context-dependent behavior across acute and chronic phases of inflammation. For example, IL-6 is included in both the Vata and Pitta sub-scores, as it serves as a *pleiotropic* cytokine that regulates transition from acute to chronic inflammation through inducing MCP-1 production and differentiation of Th17 cells (112). This overlap reflects the regulatory nature of these cytokines, serving as catalytic switches that determine the direction of the inflammatory flux. Similarly, IL-22 is included within the Vata component to reflect its role in functional plasticity acting as a pleiotropic cytokine in Th17-associated pathways (113).

The Kapha sub-score captures anti-inflammatory resilience through IL-10 promoter haplotype (rs1800896/rs1800871/rs1800872) (114), TGF-*β*1 (rs1982073) (115), IL-4 (rs2243250) (116), IL-13 (rs1800925) (117), and IL-37 (rs3811047/rs3811046) (118). IL-10 and TGF-*β*1 represent the baseline anti-inflammatory and tissue repair capacity, providing constitutional structural and immunoregulatory stability consistent with Kapha’s anabolic phenotype. The inclusion of IL-4 and IL-13 reflects their canonical role as primary inducers of M2 macrophage polarization and Th2-mediated immunity — the adaptive immune counterpart of Kapha’s oxidative metabolic phenotype — and their association with Kapha-type respiratory disorders. This underscores the Ayurvedic principle that pathological Kapha arises from the same anabolic pathways that provide homeostatic stability. The inclusion of IL-37 provides a specific marker for suppression of the IL-1 family and preservation of metabolic homeostasis (119). Overall, the proposed GII framework represents a three-dimensional projection of an individual’s complex, high-dimensional genetic inflammatory architecture, through the genomic Prakriti vector ${\vec{P}}_{G}$, through a mapping between the Vata, Pitta, and Kapha doshas to cytokine SNP alleles, as presented in **Table 1**. As we show below, one can reduce the dimensionality further by constructing a two-dimensional projection of ${\vec{P}}_{G}$ by decomposing into pro-inflammatory and anti-inflammatory components.

**Table 1:** Proposed representative mapping between doshas and cytokines for calculating prakriti genetic vector ${\vec{P}}_{G}$.

Dosha	Cytokine	SNP	rs Number	Allele	${\vec{P}}_{G}$ component
Vata	IL-6	-174 G>C	rs1800795	G (risk)	V_s_
Vata	IL-17A	-197 G>A	rs2275913	A (risk)	V_s_
Vata	MCP-1/CCL2	-2518 A>G	rs1024611	G (risk)	V_s_
Vata	IL-22	-527 G¿A	rs2227473	A (risk)	V_s_
Pitta	TNFα	-308 G>A	rs1800629	A (risk)	P_s_
Pitta	IL-1β	-511 C>T	rs16944	T (risk)	P_s_
Pitta	IL-1β	+3954 C>T	rs1143634	T (risk)	P_s_
Pitta	IFNγ	+874 T>A	rs2430561	T (risk)	P_s_
Pitta	IL-6	-174 G>C	rs1800795	G (risk)	P_s_
Kapha	IL-10	-1082 A>G	rs1800896	GCC (protective)	K_s_
Kapha	IL-10	-819 C>T	rs1800871	GCC (protective)	K_s_
Kapha	IL-10	-592 C>A	rs1800872	GCC (protective)	K_s_
Kapha	TGF-β1	Codon 10 T>C	rs1982073	C (protective)	K_s_
Kapha	IL-4	-590 C/T	rs2243250	T (functional)^a^	K_s_
Kapha	IL-13	-1055 C/T	rs1800925	T (functional)^a^	K_s_
Kapha	IL-37	-115 A>G	rs3811047	A/T (protective)	K_s_
Kapha	IL-37	Exon 2 Variant	rs3811046	A/T (protective)	K_s_

^a^Functional markers: higher allele dosage reflects greater M2/Th2-axis activation and contributes positively to K_s_.

#### 4.3.3 GII as the measure of inflammatory phenotype

To derive the inflammatory phenotype of an individual from the normalized genetic Prakriti vector ${\hat{P}}_{G}$, we consider as the reference a state of dosha balance described in Ayurveda, a *Sama* state, as defined in Equation 9 that constitutes the unit vector $\hat{S}$ along the symmetric diagonal of ${\mathbb{R}}^{3}$:

(9) $$\hat{S}=\frac{1}{\sqrt{3}}[\begin{array}{c} 1\\ 1\\ 1 \end{array}]$$

Relative to $\hat{S}$, we decompose ${\hat{P}}_{G}$ into two orthogonal components as follows. The component of ${\hat{P}}_{G}$ along $\hat{S}$ is given by Equation 10 as:

(10) $${\hat{P}}_{p\text{roj}}=({\hat{P}}_{G}\cdot \hat{S})\hat{S}$$

The component of ${\hat{P}}_{G}$ orthogonal to $\hat{S}$ constitutes the residual drift vector $\vec{D}$ that captures constitutional deviation from $\hat{S}$ as given by Equation 11:

(11) $$D\;={\hat{P}}_{G}-{\hat{P}}_{\text{proj}}=[\begin{array}{c} e_{V}\\ e_{P}\\ e_{K} \end{array}],$$

where *e_V_*, *e_P_*, and *e_K_* are the residual components of ${\hat{P}}_{G}$ after removing its projection onto $\hat{S}$. By construction, for an individual with *Sama* Prakriti, the angle between vectors ${\hat{P}}_{G}$ and $\hat{S}$ is expected to approach 0°, which implies $D\;\approx \vec{0}$. In other Prakriti types, the angular deviation *θ* between ${\hat{P}}_{G}$ and $\hat{S}$ quantifies dosha imbalance as given by Equation 12:

(12) $$\theta =\cos^{-1}({\hat{P}}_{G}\cdot \hat{S})\in [0^{{}^{\circ}},90^{{}^{\circ}}].$$

However, *θ* does not directly provide information on whether the imbalance is of pro-inflammatory type or of anti-inflammatory type. It only characterizes the constitutional dosha imbalance relative to the reference $\hat{S}$. To accomplish inflammatory phenotype stratification, the drift vector $\vec{D}$ can be decomposed into a pro-inflammatory component in the Vata–Pitta plane and an orthogonal anti-inflammatory component along the Kapha axis as in Equation 13:

(13) $$D_{\text{pro}}=e_{V}+e_{P},\;\;D_{\text{anti}}=e_{K}.$$

We define the GII, using Equation 14 as the difference between the pro-inflammatory component *D*_pro_ and the anti-inflammatory component *D*_anti_ of the drift vector, quantifying the amplitude and direction of inflammatory imbalance of an individual:

(14) $$\text{GII}=D_{\text{pro}}-D_{\text{anti}}=e_{V}+e_{P}-e_{K}.$$

The sign of GII (see Equations 15–17) characterizes the inflammatory phenotype:

(15) GII>0 :pro−inflammatory phenotype,

(16) GII<0 :anti−inflammatory phenotype,

(17) GII≈0 :balanced inflammatory phenotype.

When *θ* = 0° and GII = 0, the profile corresponds to Sama Prakriti, suggesting a balanced constitutional state. The sign of GII indicates the direction of inflammatory polarity: positive GII denotes a proinflammatory phenotype, negative GII corresponds to an anti-inflammatory or tissue-protective phenotype. Importantly, the magnitude of GII reflects the strength of the inflammatory phenotype: larger positive values are associated with higher susceptibility to inflammaging and innate immune activation, while negative values indicate relatively lower susceptibility. Finally, it is notable that the inflammatory phenotyping uses as reference the Sama vector $\hat{S}$, an Ayurvedic construct; its use is interpretively meaningful only if ${\hat{P}}_{G}$ constitutes a valid genomic proxy for ${\hat{P}}_{A}$. This concordance is validated in Section 4.5.

### 4.4 Epigenetic age acceleration as an observable of inflammaging

To evaluate whether differences in baseline inflammatory tone across Prakriti types translate into measurable differences in biological aging, we propose a Prakriti-stratified *e*_AA_ analysis using epigenetic clocks that measure the progress of biological aging as a cumulative departure from chronological age. Since the hypothesis concerns the cumulative integration of genetic and epigenetic influences over the lifespan, the appropriate instruments are those demonstrating high heritability and longitudinal stability reflecting genetic rather than a purely environmental signal, and that measure average pace of aging. PCGrimAge (85) can be used as the primary clock for this purpose, due its longitudinal stability being attributable to genetic rather than environmental factors (85). PCPhenoAge can be employed as a complementary measure, with particular relevance to the inflammaging context through its incorporation of NF-*κ*B and inflammatory pathway CpGs, and a heritability of 0.70 in twin studies (85). Together, these two clocks provide a robust and heritable integrated readout of cumulative inflammatory burden, well-suited to detecting Prakriti-based differences in inflammaging trajectories.

### 4.5 Theoretical consistency, validation and statistical justification

#### 4.5.1 Theoretical consistency of GII

To verify the internal consistency of the GII framework, we computed *θ* and GII for four extreme constitutional types — Sama, Vata, Pitta, and Kapha — using hypothetical normalized genetic Prakriti vectors ${\hat{P}}_{G}$, as shown in **Table 2**. The Sama case yields *θ* = 0° and GII = 0, correctly identifying a balanced inflammatory phenotype. Both Vata and Pitta dominance produce *θ >* 0° and GII *>* 0, indicating a pro-inflammatory phenotype consistent with their tendency for heightened inflammatory responses. In contrast, Kapha dominance produces *θ >* 0° but GII *<* 0, reflecting an anti-inflammatory or tissue-protective phenotype. These examples confirm that *θ* quantifies the magnitude of constitutional dosha imbalance, while GII determines the inflammatory phenotype and its strength.

**Table 2:** Theoretical worked examples of GII calculation for extreme constitutional types.

Case	${\hat{P}}_{G}$ = (Vs,Ps,Ks)	θ	GII	Inflammatory Phenotype
Sama (balanced)	(0.577,0.577,0.577)	0.0°	0.000	Balanced
Vata dominant	(0.896,0.398,0.199)	30.5°	+0.597	Pro-inflammatory
Pitta dominant	(0.518,0.830,0.207)	26.1°	+0.622	Pro-inflammatory
Kapha dominant	(0.424,0.318,0.848)	23.4°	−0.636	Anti-inflammatory

#### 4.5.2 Validation and statistical justification

To validate the central assumption that the genomic Prakriti vector ${\hat{P}}_{G}$ constitutes a valid proxy for the Ayurvedic Prakriti vector ${\hat{P}}_{A}$, we propose two complementary measures. First, for each Dosha axis, the Spearman rank correlation between genomic and Ayurvedic scores is computed:


$$\rho_{V}=\text{corr}(V_{A},V_{s}),\;\;\rho_{P}=\text{corr}(P_{A},P_{s}),\;\;\rho_{K}=\text{corr}(K_{A},K_{s}).$$

Significant positive correlations (*p <* 0.05) across all three axes would support concordance. Second, the angle between the normalized Ayurvedic Prakriti vector and normalized genomic Prakriti vector vector calculated using Equation 18:

(18) $$\alpha =\cos^{-1}({\hat{P}}_{A}\cdot {\hat{P}}_{G})$$

provides a per-individual concordance measure, with a small population-level *α* confirming directional agreement between genotypic and phenotypic characterization. To test statistical significance of HypothesisI – pro-inflammatory drift in Vata and Pitta individuals and an anti-inflammatory drift in Kapha – the Wilcoxon signed-rank test can be used separately for each Prakriti group to assess whether the median GII differs significantly from zero. For validating Hypothesis-II – *e*_AA_(Vata) *> e*_AA_(Pitta) *> e*_AA_(Kapha) *> e*_AA_(Sama) – the Jonckheere–Terpstra test can be employed, as it is specifically designed for monotonic trends across ordered groups. These tests can be implemented in R using the wilcox.test() function for the Wilcoxon signed-rank test and the jonckheere.test() function from the clinfun package for the Jonckheere–Terpstra test.

## 5 Discussion

Understanding interindividual variations in susceptibility to inflammaging as a function of the genetic makeup of an individual is important for developing personalized and preventive medical strategies in managing chronic inflammatory disorders. However, extensive genome sequencing and analyzing the generated data to understand the underlying molecular mechanisms behind such variations is a tedious and challenging task. The emerging field of ayurgenomics takes a complementary approach by attempting to derive genotype-phenotype correlations through the concept of Ayurveda Prakriti, the constitutional phenotype of an individual. Such correlations require identifying genes involved in key regulatory processes and mechanisms that differ between Prakriti. The conventional Ayurvedic knowledge system has evolved in India as an experiential form of medicine, identifying metabolism and immune responses as two critical manifestations of Prakriti. Interestingly, research over the last three decades in the areas of inflammaging, trained immunity, and metaflammation has also converged on the intricate link between metabolism and immunity through the concept of immunometabolic reprogramming.

In the present hypothesis paper, we started with the following pressing questions (1): Do variations in metabolism and immune responses have a genetic origin? (2) If so, would this mean differences in baseline genetic inflammatory tone? (3) Is it possible to identify such differences using a phenotypic stratification based on Ayurveda Prakriti? These questions naturally led us to integrate insights from all the aforementioned diverse fields, resulting in the two hypotheses presented in Section 5. Our hypotheses culminate in the proposal that the three doshas may correspond to distinct functional tendencies within inflammatory regulation. Pitta dosha can be interpreted as the initiator of inflammation, triggering a rapid immune reflex that induces an acute inflammatory innate immune response. Kapha dosha is hypothesized to reflect processes that resolve inflammation and perform tissue repair. This is consistent with the robust adaptive immune responses observed in Kapha Prakriti individuals (100). Vata dosha may function analogously to a regulatory mechanism that balances pro-inflammatory and anti-inflammatory networks. Within the hypotheses presented here, inflammaging can be understood as a failure of regulatory switching due to aggravation of Vata dosha with aging. The regulatory role of Vata finds preliminary evidence in clinical studies of RA (104) and observations of maximum drop in parasympathetic activity under orthostatic stress in Vata individuals compared to other Prakriti types (102).

Within the mathematical framework proposed here, interindividual differences in susceptibility to inflammaging can be quantified in terms of GII that measures the amplitude and direction of inflammatory imbalance in an individual. Unlike isolated inflammation markers such as circulating CRP, IL-6, IL-1*β*, and TNF-*α* levels, which are influenced by transient intrinsic and extrinsic factors, the GII captures constitutional genetic architecture to quantify baseline inflammatory susceptibility. Moreover, GII complements existing composite indices, such as INFLA-score (120), which integrates information from multiple circulating inflammatory markers to quantify current inflammatory state.

### 5.1 Limitations and confounding effects

The present hypothesis framework has several limitations that should be acknowledged. First, the interpretation of dual Prakriti (Dwandwaja) types is not considered in this framework. The Ayurvedic clinical management of Dwandwaja Dosha disorders is considered complex due to the coexistence of Doshas with opposing qualitative attributes (Guna) and functional actions (Karma). Within the inflammatory framework proposed here, it can be speculated that Vata-Pitta individuals might have a tendency for persistent innate immune activation, while Vata-Kapha individuals might show a contrasting behavior. However, it must be emphasized that there is currently no direct experimental evidence for these interpretations. Second, with respect to confounding factors between inflammaging and biological aging, it was observed that some centenarians achieve extraordinary longevity despite suboptimal dietary or physical activity patterns (121) highlights the presence of confounding variables, such as protective genetic backgrounds, effective immune regulation, and advantageous metabolic characteristics. Third, the relationship between epigenetic age acceleration and biological aging is complicated by the existence of epigenetic erasers — enzymes such as TET proteins and KDM family histone demethylases that actively reverse epigenetic modifications (122, 123). Recent evidence suggests that biological age can decrease under certain conditions, such as stress recovery (124). Our framework assumes that *e*_AA_ primarily captures cumulative inflammatory burden, but the contribution of epigenetic reversibility — and whether such reversibility differs across Prakriti types — remains unknown. Finally, an important limitation is that GII being a low-dimensional projection of the Prakriti vector, distinguishes pro-inflammatory and antiinflammatory predispositions but does not differentiate between Vata- and Pitta-dominant constitutions. A potential future refinement requires introducing an additional azimuthal angle *ϕ* within the Vata-Pitta plane, enabling models for genetic baseline of epigenetic age acceleration of the form: *g*(*B*) = *f*(GII) + *h*(*ϕ*) in Equation 2.

## Data Availability

The original contributions presented in the study are included in the article/supplementary material. Further inquiries can be directed to the corresponding authors.
